# Effects of PGK1 on immunoinfiltration by integrated single-cell and bulk RNA-sequencing analysis in sepsis

**DOI:** 10.3389/fimmu.2024.1449975

**Published:** 2024-12-06

**Authors:** Yu Liu, Weijie Li, Lei Lei, Yaoliang Zhou, Mingcheng Huang, Yide Li, Xiaoying Zhang, Yingyu Jiang, Haiqi Wu, Zhihua Zheng, Kongyang Ma, Chun Tang

**Affiliations:** ^1^ Department of Nephrology, Center of Kidney and Urology, The Seventh Affiliated Hospital, Sun Yat-sen University, Shenzhen, China; ^2^ Centre for Infection and Immunity Studies, School of Medicine, The Sun Yat-sen University, Shenzhen, China; ^3^ Emergency and Disaster Medical Center, The Seventh Affiliated Hospital, Sun Yat-sen University, Shenzhen, China; ^4^ Department of Critical Care Medicine, The Seventh Affiliated Hospital, Sun Yat-sen University, Shenzhen, China; ^5^ Health Management Center, The Seventh Affiliated Hospital, Sun Yat-sen University, Shenzhen, China; ^6^ Department of Renal Rheumatology and Immunology, The People’s Hospital of Hezhou, Hezhou, China

**Keywords:** sepsis, PGK1, immune infiltration, single-cell RNA-sequencing, biomarker

## Abstract

**Background:**

Sepsis, a life-threatening organ dysfunction caused by a dysregulated immune response to infection, remains a significant global health challenge. Phosphoglycerate kinase 1 (PGK1) has been implicated in regulating inflammation and immune cell infiltration in inflammatory conditions. However, the role of PGK1 in sepsis remains largely unexplored.

**Methods:**

Four microarray datasets and a high throughput sequencing dataset were acquired from GEO database to reveal the PGK1 expression in patients of sepsis. Quantitative real-time PCR and western blotting was then used to validate the PGK1 level. Additionally, microarray and single-cell RNA sequencing data integration, including gene set enrichment analysis (GSEA), KEGG and GO functional enrichment analysis, immune infiltration analysis, and single-cell sequencing analysis, were performed to elucidate the role of PGK1 in sepsis.

**Results:**

Our results revealed a significant upregulation of PGK1 in sepsis patients, with the area under the ROC curve (AUC) exceeding 0.9 across multiple datasets, indicating PGK1’s strong potential as a diagnostic biomarker. Notably, PGK1 was enriched in key immune-related pathways, including the TNF signaling pathways, and leukocyte transendothelial migration, suggesting its involvement in immune regulation. Furthermore, PGK1 expression showed a positive correlation with the levels of inflammatory mediators CXCL1, CXCL16, and the chemokine receptor CCR1. In terms of immune cell infiltration, PGK1 was positively correlated with naive B cells, resting memory CD4 T cell, gamma delta T cells, M0 macrophages, eosinophils and negatively correlated with plasma cells, CD8 T cells, activated memory CD4 T cell, Tregs, activated dendritic cells.

**Conclusions:**

This study concluded that PGK1 served as a novel diagnostic biomarker for sepsis, with potential implications for prognosis and immune regulation. The significant upregulation of PGK1 in sepsis patients and its association with immune-related pathways and cell types highlight its potential role in the pathogenesis of sepsis.

## 1 Introduction

Sepsis is defined as life-threatening organ dysfunction caused by a dysregulated immune response to infection (1). The Third International Consensus Definitions for Sepsis and Septic Shock (Sepsis-3), updated in 2016, emphasized the critical role of organ dysfunction in the diagnosis of sepsis (2). Sepsis represents a major global health challenge, affecting an estimated 48.9 million people and causing 11 million deaths annually, which accounts for nearly 20% of all global fatalities (3). The pathophysiology of sepsis is complex and multifactorial, involving interactions between the host immune system, the inflammatory response, and the infecting microorganisms (4). The host’s response to infection can lead to a systemic inflammatory response syndrome (SIRS), which may progress to severe sepsis and septic shock (5). The concept of ‘early-onset sepsis (EOS) and late-onset sepsis (LOS)’ has shaped reporting of neonatal sepsis, and strengthening the reporting of observational studies in epidemiology for newborn infection (STROBE-NI) reporting guidelines were recommended to move toward reporting of age of onset as a continuous variable (6). Consequently, understanding the intricate pathophysiological mechanisms of sepsis and identifying potential biomarkers are crucial for developing targeted therapies and improving clinical outcomes.

In sepsis, the immune system is essential for fighting pathogens, yet its excessive activation paradoxically causes tissue and organ damage (7). The initial response to infection is crucial for pathogen clearance, however, this response spirals into a SIRS (8), characterized by an excessive release of pro-inflammatory cytokines such as interleukin-1 (IL-1), interleukin-6 (IL-6), and tumor necrosis factor-alpha (TNF-α) (9). Innate immune cells, including neutrophils, macrophages, dendritic cells, and natural killer T cells, are critical in SIRS during sepsis development and subsequent organ damage (10). Neutrophils, for instance, exacerbated collateral damage by releasing reactive oxygen species (ROS) and forming neutrophil extracellular traps (NETs) (11). Recent study reported that neutrophil activation related proteins LCN2 and RETN were potentially as targeted biomarkers for predicting COVID-19 and sepsis-induced ARDS in elderly patients (12). The uncontrolled activation and differentiation of T cells resulted in heightened cytokine production, leading to excessive inflammation (13). As sepsis progresses, it transitions into an immunosuppressive phase characterized by lymphocyte apoptosis and a reduction in protective cytokines, a state termed compensatory anti-inflammatory response syndrome (CARS) (14). This overactive immune response directly causes organ injury through inflammation, oxidative stress, and cell apoptosis (15).

Phosphoglycerate kinase 1 (PGK1), an enzyme central to glycolysis, catalyzes the conversion of 1,3-bisphosphoglycerate to 3-phosphoglycerate, a reaction that is not only instrumental in ATP production but also essential for the cellular energy supply (16). PGK1 was pivotal in the proliferation of tumor cells (17), and its downregulation significantly diminished their glycolytic activity, curtailing tumor cell proliferation and tumorigenesis (18). During the activation phase of the adaptive immune response, glycolysis was upregulated, underscoring significance of PGK1 in the metabolic pathways that fueled T cell activation (19, 20). Within the tumor microenvironment, PGK1 was observed at heightened levels in both tumor cells and immunosuppressive T cells, where it played a role in fostering an immunosuppressive milieu (21). In lung adenocarcinoma, PGK1 expression interacted with various immune and inflammatory signaling pathways. Additionally, PGK1 contributed to the recruitment of key immune cells such as dendritic cells, macrophages, and neutrophils. Importantly, it was notably elevated in immunosuppressive cell populations, including M2 macrophages, regulatory T cells (Tregs), and exhausted T cells (21). Additionally, PGK1 was believed to influence neutrophil activation (22). These studies suggested that PGK1 interacted with immune cells through diverse mechanisms, potentially modulating immune responses and implicating a role for PGK1 in the immunopathology of sepsis. However, the specific role of PGK1 in the dysregulated immune response in sepsis remains unclear.

In this study, we aimed to investigate the diagnostic potential of PGK1 in sepsis by examining its expression in patients with sepsis and evaluating its correlation with immune cell. We posited that PGK1 may serve as a sensitive and specific biomarker for the diagnostic assessment of sepsis, offering a novel perspective on the interplay between immunity and organ dysfunction in this complex clinical scenario.

## 2 Materials and methods

### 2.1 Subjects and samples

Human subjects were enrolled in this study from the Seventh Affiliated Hospital of Sun Yat-sen University. All sepsis patients fulfilled the diagnostic criteria of The Third International Consensus Definitions for Sepsis.[2] Informed consents were acquired from all participants. This study was approved by the Ethics Committee of the Seventh Affiliated Hospital of Sun Yat-sen University (Certificate Number: KY-2020-070-01) and conducted in accordance with the Declaration of Helsinki. Peripheral whole blood from sepsis patients and healthy volunteers were collected using EDTA anticoagulated tubes. Then peripheral blood mononuclear cells (PBMCs) were separated with Lymphocyte Separation Medium (Solarbio, P8610) according to manufacturer’s instructions.

### 2.2 RNA extraction and quantitative real-time PCR

Total RNA was extracted from PBMCs using NcmSpin total RNA Kit (NCM Biotech, M5105) and reverse transcribed into cDNA using *Evo M-MLV* RT Mix Kit with gDNA Clean for qPCR Ver.2 (Accurate Biology, AG11728). PGK1 mRNA expression was quantified using quantitative real-time PCR (qPCR). qPCR was conducted on Applied Biosystems QuantStudio 3 (Thermo Fisher) using SYBR Green Premix *Pro Taq* HS qPCR Kit (Accurate Biology, AG11701) as recommended by the manufacturer. The relative PGK1 mRNA expression was normalized to the GAPDH. Primer sequences: PGK1: forward 5’-GACCGAATCACCGACCTCTC-3’ reverse 5’-AGCAGCCTTAATCCTCTGGT-3’; GAPDH: forward 5’-AGAAGGCTGGGGCTCATTTG-3’ reverse 5’-AGGGGCCATCCACAGTCTTC-3’.

### 2.3 Western Blotting

Homogenize the PBMCs first in the lysis buffer (CoWin Biosciences, CW2333S) to extract proteins and determine protein concentration using a BCA assay (NCM Biotech, WB6501). Load equal amounts of protein onto an SDS-PAGE gel and separate by electrophoresis. Transfer the separated proteins onto the PVDF membrane using a semi-dry transfer apparatus. Block the membrane with 5% non-fat milk to prevent non-specific binding. Incubate with primary antibodies for anti-PGK1 (1:1000, Proteintech, 17811-1-AP) and anti-GAPDH (1:1000, Proteintech, 60004-1-Ig) overnight at 4°C. Wash and then incubate with secondary antibodies conjugated to horseradish peroxidase at room temperature, including anti-rabbit IgG-HRP antibody (1:2000, ABclonal, AS014) or anti-mouse IgG-HRP antibody (1:2000, ABclonal, AS003). Develop the blot using chemiluminescent substrate and visualize the bands on an X-ray film or digital imaging system. Use the ultra-high sensitivity ECL kit (GLPBIO, GK10008) and visualize the bands on Bio-Rad ChemiDoc XRS+ Systetem. Image J was used to quantitatively analyze the relative expression of the bands.

### 2.4 Data availability

Dataset profiles that explored the gene expression were obtained from the Gene Expression Omnibus (GEO) public database (http://www.ncbi.nlm.nih.gov/geo/). GSE28750, GSE57065, GSE65682, GSE95233, GSE154918 consisted of gene expression in PBMCs or whole blood from sepsis patients and healthy controls (HCs). Single-cell RNA sequencing (scRNA-seq) data obtained from GSE167363. The sample type, experiment type, and sample size of the datasets used in this research were summarized in **Table 1**. Sepsis patients from GSE167363 were divided into survivor (S), nonsurvivor, late-stage sepsis (NS LS) with fatality within 24 h, and nonsurvivor, early-stage sepsis (NS ES) with fatality within 30 days.

**Table 1:** Overview of the sample type, experiment type, and sample size of the datasets used in this research.

Dataset	GSE28750	GSE57065	GSE65682	GSE95233	GSE154918	GSE167363
Sample	PBMCs	PBMCs	Whole-blood leukocytes	PBMCs	PBMCs	PBMCs
Experiment Type	Array	Array	Array	Array	RNA-seq	scRNA-seq
Sample Size	Sepsis: 10 HC: 20	Sepsis: 82 HC: 25	Sepsis: 760 HC: 42	Sepsis: 102 HC: 22	Sepsis: 65 HC: 40	Sepsis: 10 HC: 2

### 2.5 Receiver operating characteristic curve

The receiver Operating Characteristic (ROC) curve is a commonly used tool to assess the accuracy of diagnostic tests. The ROC curve assessed the diagnostic performance of the test by plotting the relationship between true case rate (sensitivity) and false positive case rate (1-specificity). In gene expression data analysis, ROC curves can be used to evaluate the efficacy of specific genes or genomes in cooperating as disease state classifiers. We used the pROC R package to generate the ROC curve and calculate the Area Under the Curve (AUC).

### 2.6 Kaplan-Meier survival curve analysis

All survival data were analyzed using the statistical software R, specifically the Kaplan-Meier analysis using the survival package. Differences in survival curves were compared using the Log-rank test to assess differences in survival between groups.

### 2.7 Screening of differentially expressed genes and PGK1-coexpressed genes

Based on the results of the gene expression matrix of the dataset, we determined whether there was a difference between the two independent groups using the DESeq2 and limma packages, which could perform the non-parametric statistical method of the Mann-Whitney U test. High and low PGK1 expression groups were defined based on mean PGK1 expression of sepsis patients. Patients with PGK1 expression above the mean PGK1 expression were classified as the high expression group, while patients with PGK1 expression below the mean PGK1 expression were classified as the low expression group. We identified differentially expressed genes (DEGs) between high_PGK1 and low_PGK1 group with a *P* adjust < 0.05 and a log (fold change) >1 or log (fold change) <−1 as the cut-of criteria. By using Pearson’s correlation test, we screened the PGK1-coexpressed genes with the threshold point of absolute r > 0.4 and P adjust < 0.05. And we used the ggplot2 package to draw volcano plots and heat maps.

### 2.8 Gene set enrichment analysis

To assess whether there was a statistically significant difference in the expression of a particular gene set between patients with sepsis and healthy people, the method of gene set enrichment analysis (GSEA) was performed. In short, Gene expression data were first sorted based on the association between genes and phenotypes, and then an enrichment score (ES) for a predefined gene set was calculated to determine whether the gene set is rich at the top and bottom. The gseKEGG(), gseGO() functions were used in the clusterProfiler package to do GSEA enrichment analysis.

### 2.9 Gene set variation analysis

In this dataset, we used Gene Set Variation Analysis (GSVA) to assess differences in gene set expression on a single sample, as an unsupervised method to estimate changes in gene set activity in a sample. In this study, we used the GSVA package to obtain the gene set enrichment score for each sample to explore how gene set activity in sepsis and healthy people correlated to specific biological pathways.

### 2.10 Immune infiltration analysis

To estimate the relative proportions of immune cell types in GSE65682, we used the CIBERSORTx tool with the following parameter settings. Our standardized bulk RNA-seq data served as the mixture file. We selected the “Impute Cell Fractions” analysis module to estimate cell type proportions. Batch correction mode was enabled (B-mode) to adjust for batch effects between the reference matrix and our mixture samples. The number of permutations was set to 1000 to achieve robust statistical inference. Visualization was performed using the ggplot2 package in R to generate bar plots and heatmaps of cell composition.

### 2.11 Single-cell RNA sequencing analysis

First, the Seurat package was used to process the feature-cell matrix to generate the original feature matrix, and then cell screening was performed to remove cells with abnormal gene expression (less than 200 or more than 3000 genes) and high mitochondrial gene expression (>10%). FeaturePlot and VlnPlot were used to visualize key quality control indicators, including the number of genes, UMI number, and mitochondrial gene percentage of each cell. And we used FindVariableFeatures function to select 2,000 highly variable genes. Then, LogNormalize normalization of the data was performed to ensure that the total number of molecules in each cell was 10,000, and then the genes with the most variable expression were identified to select variant features. Then, principal component analysis (PCA) was performed to reduce the data dimension, and then the PCA results were batch corrected using the Harmnoy algorithm with key parameters set to: max.iter.harmony = 20, lambda = 1, theta = 2. After Harmony integration, t-SNE and UMAP dimensionality reduction analysis were performed again based on PCA coordinates, focusing on the first 30 principal components, and, we performed gene-resolution clustering analysis to identify cell subpopulations by constructing a KNN graph using the features based on Harmony correction, applying the FindNeighbors function with k.param=15. Next, we used the FindClusters function with resolution = 0.8 to identify distinct cell clusters. At the same time, cell populations were identified and annotated according to different marker genes. Finally, the FindMarkers function was used to perform differential gene expression analysis on cell subpopulations.

### 2.12 Statistical analysis

All data were presented as mean ± standard error of the mean (SEM). The statistical analyses were performed using GraphPad Prism 7.0. A two-tailed Student t-test and the Mann-Whitney U test were used to analyze the statistical significance between the two groups. Kaplan-Meier tests were used under the log-rank algorithm for survival analysis. The Pearson chi-squared test was used to analyze the correlation between PGK1 and immune characteristic variables. For multiple tests, we applied the Benjamini-Hochberg (BH) method to adjust the *P*-value. Statistical significance was denoted as *P < 0.05, **P < 0.01, ***P < 0.001.

## 3 Results

### 3.1 PGK1 mRNA expression was obviously elevated in sepsis

**Figure 1:**
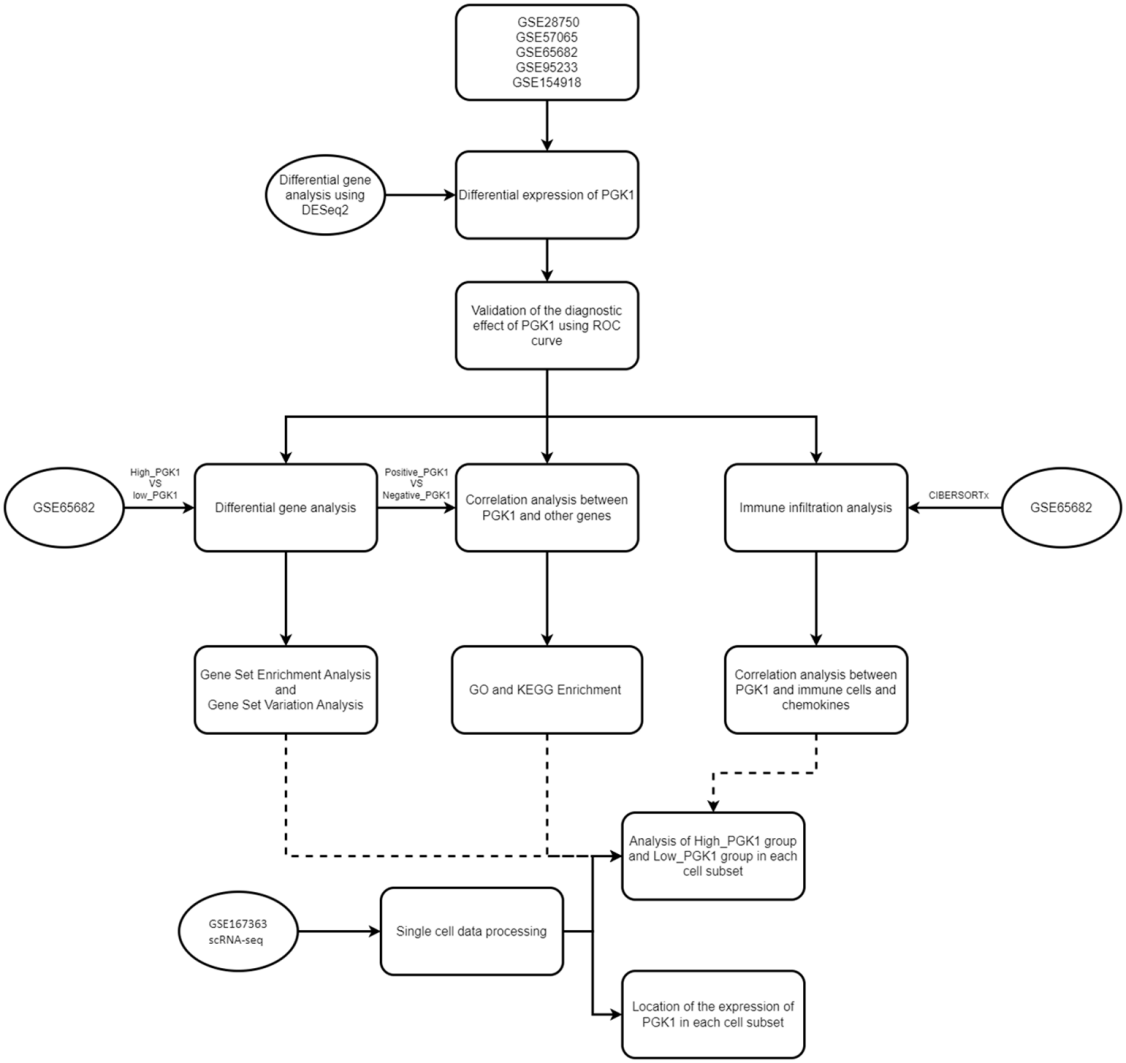
Flowchart of overall design in this study. A flow chart displaying this study design and process.

**Figure 2:**
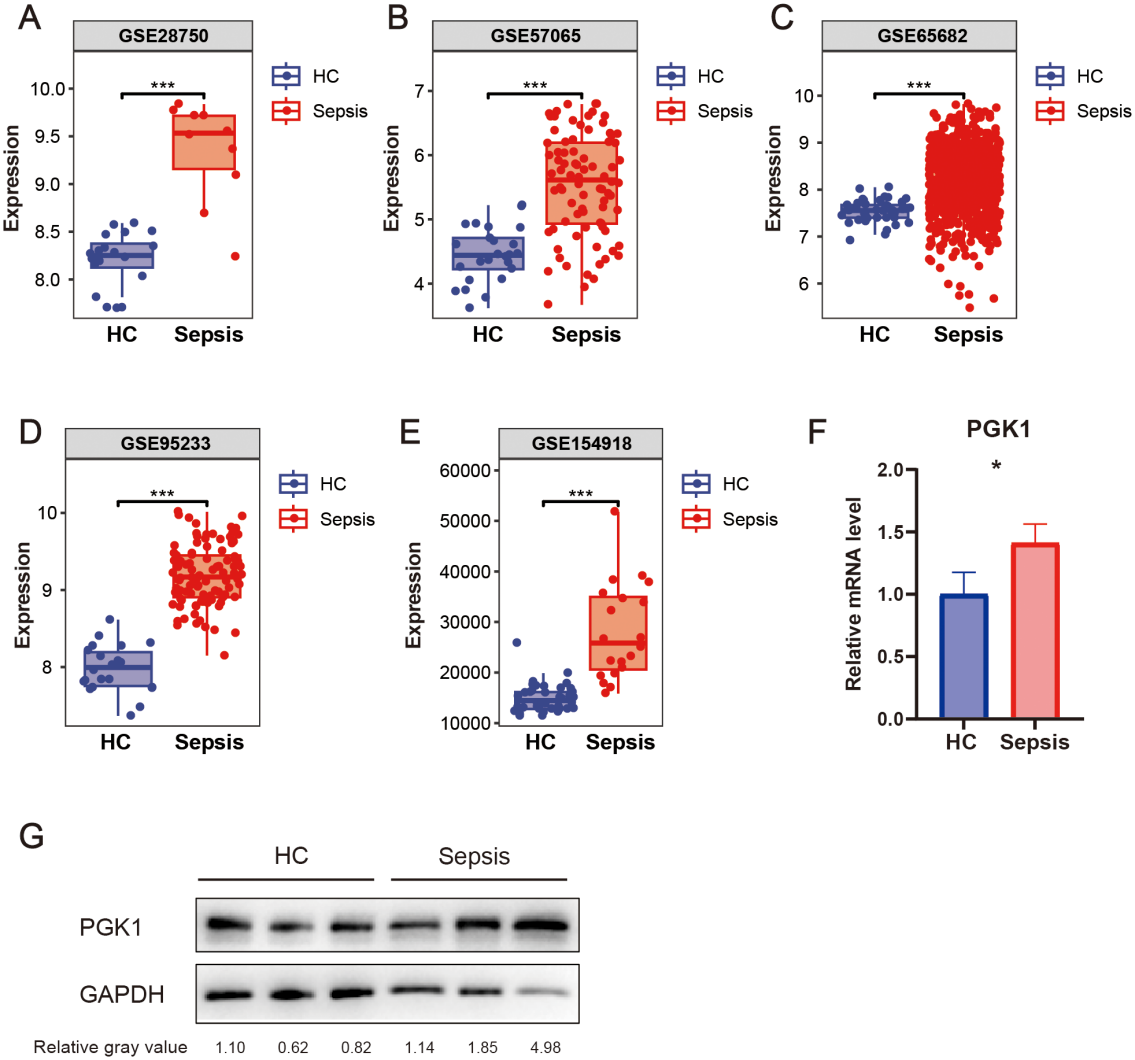
Expression level of PGK1 in sepsis. PGK1 mRNA levels in peripheral blood mononuclear cells (PBMCs) of sepsis patients and healthy people in **(A)** GSE28750, **(B)** GSE57065, **(C)** GSE65682, **(D)** GSE95233, and **(E)** GSE154918. **(F)** PGK mRNA levels in PBMCs of sepsis patients and healthy people were examined by qRT-PCR. **(G)** PGK protein levels in PBMCs of sepsis patients and healthy people were examined by western blotting. *P < 0.05, ***P < 0.001.

The bioinformatic analysis scheme of our study was shown in **Figure 1**. We used multiple GEO datasets (GSE28750, GSE57065, GSE65682, GSE95233, and GSE154918) to explore the different expression of PGK1 between healthy people and sepsis patients. The results showed that compared to the healthy controls, the expression of PGK1 mRNA was obviously increased in sepsis cohort (**Figures 2A–E**). Moreover, we verified the elevated PGK1 mRNA levels in our sepsis patients by qPCR (**Figure 2F**). We also observed that PGK1 protein showed an increased trend in sepsis patients compared to healthy controls (**Figure 2G**). These data suggested that sepsis patients presented the significantly abnormal level of PGK1.

### 3.2 PGK1 possessed a favorable diagnostic value in sepsis

**Figure 3:**
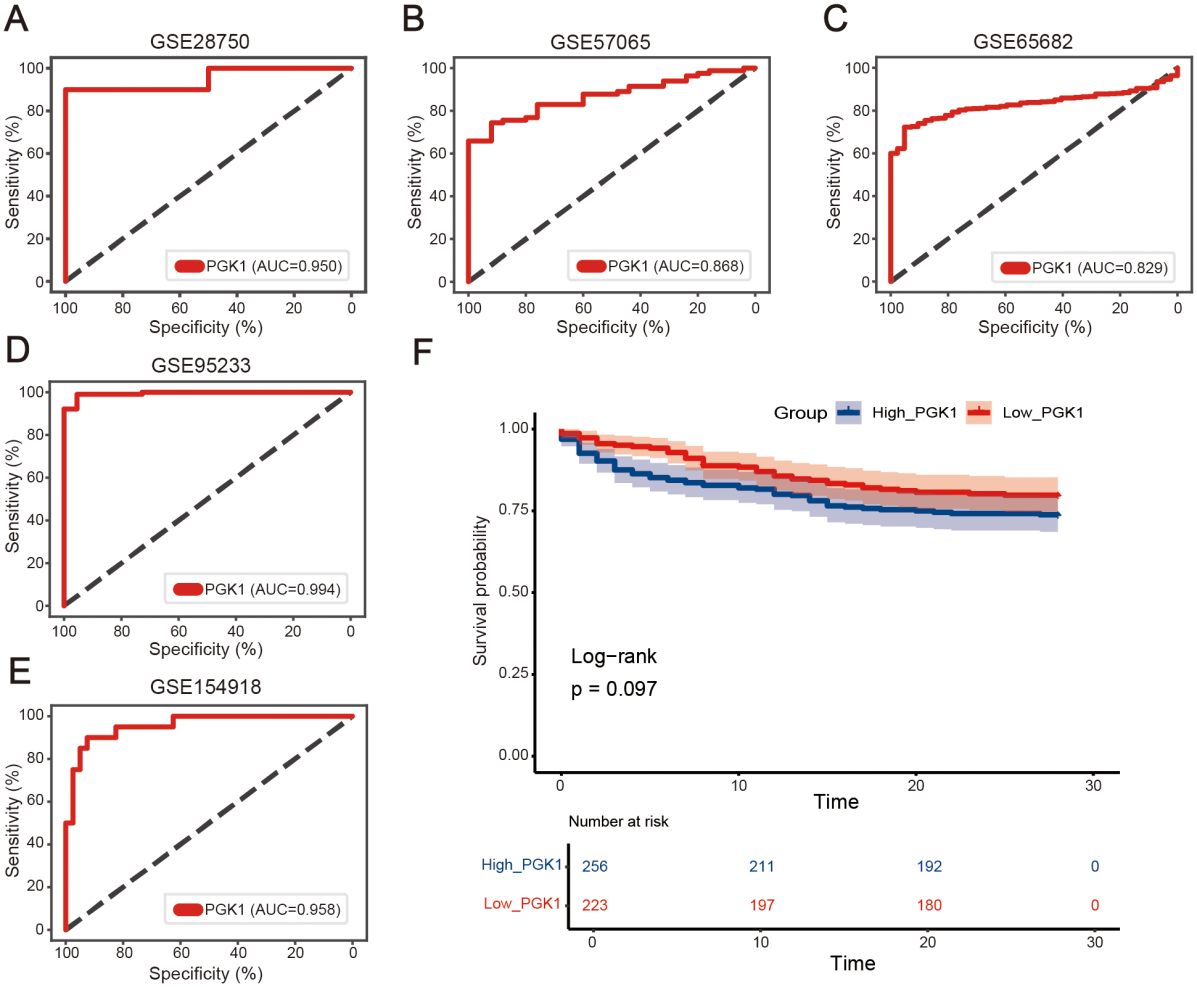
Diagnostic performances of PGK1 in sepsis. Receiver operating characteristic (ROC) curves of PGK1 expression in sepsis diagnosis in **(A)** GSE28750, **(B)** GSE57065, **(C)** GSE65682, **(D)** GSE95233, and **(E)** GSE154918 dataset. **(F)** Overall survival of high_PGK1 and low_PGK1 sepsis patients analyzed in the Kaplan-Meier plotter database with the datasets GSE65682 (n = 479).

To determine the diagnostic effectiveness of PGK1 in sepsis, the GSE28750 dataset was first used as the training dataset. The area under the ROC curve (AUC) of PGK1 to significantly distinguish between the normal group and the sepsis group was 0.950 (**Figure 3A**). Next, four datasets (GSE57065, GSE65682, GSE95233, and GSE154918) were employed to verify the results. The AUC values of the PGK1 in the four datasets were 0.868, 0.829, 0.994, and 0.958, respectively, similar to the result from the GSE28750 dataset (**Figures 3B–E**). In short, PGK1 had a good performance in the diagnosis of sepsis.

The GSE65682 dataset was used to explore the correlation between PGK1 and the prognosis in sepsis, and the sepsis patients were divided into high PGK1 group and low PGK1 group according to the median value of PGK1. Unfortunately, kaplan-Meier analysis showed that the two groups exhibited no statistically significant difference in overall survival (OS) (*P*=0.097, **Figure 3F**), though patients with high PGK1 expression may present a poorer prognosis trend.

### 3.3 PGK1 was enriched in immune-related pathways in sepsis

**Figure 4:**
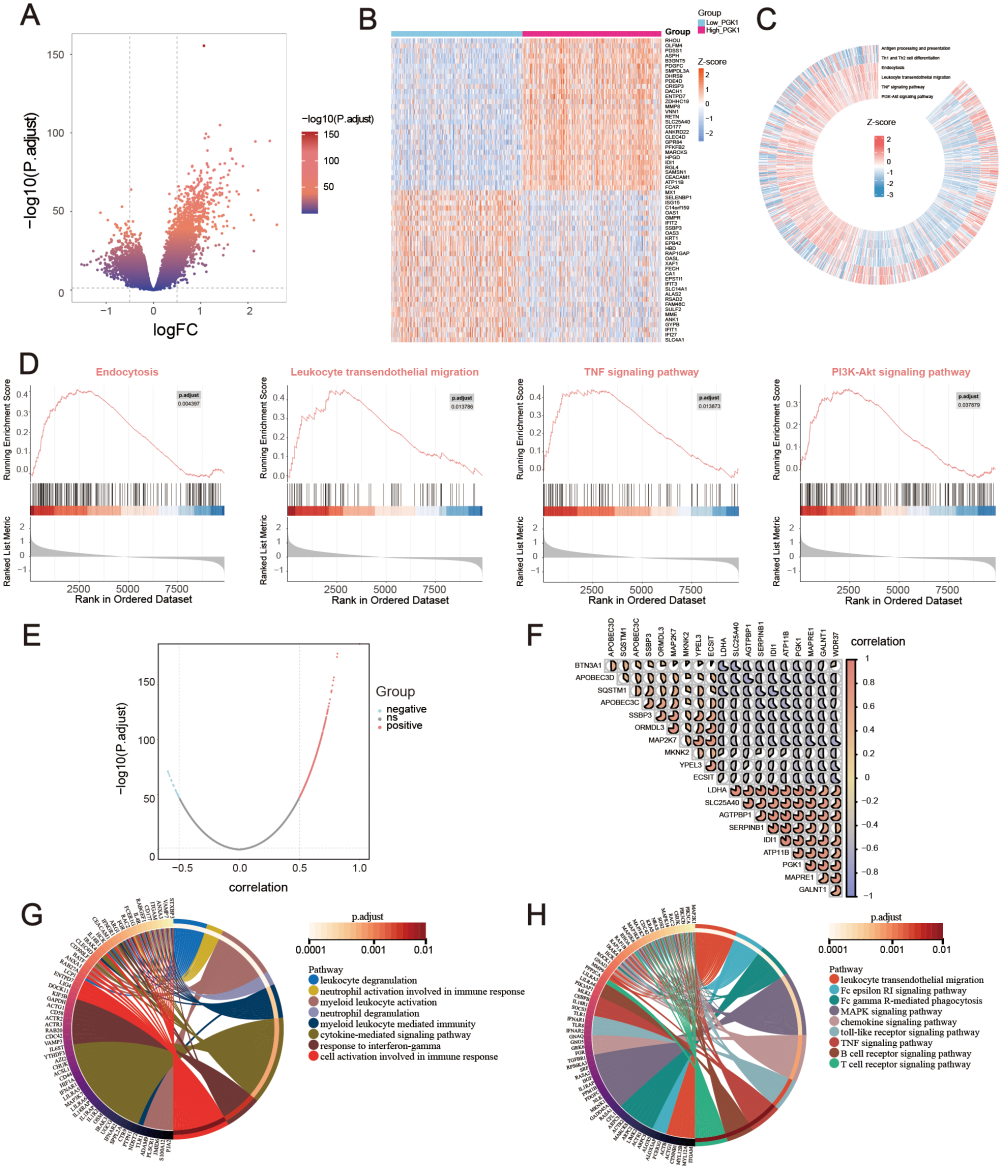
Functional exploration of PGK1 in sepsis. **(A)** Volcano plot and **(B)** Heatmap for differentially expressed genes (DEGs) between high_PGK1 and low_PGK1 sepsis patients. **(C, D)** Gene set enrichment analysis (GSEA) and Gene Set Variation Analysis (GSVA) for the signaling pathways enrichment in different groups. **(E)** Volcano plot for PGK1-coexpressed genes in sepsis. **(F)** Correlation heatmap of top 20 PGK1-coexpressed genes, the pie size indicates the degree of correlation. Circos plot for GO **(G)** and KEGG **(H)** function enrichment analysis.

Based on GSE65682 with large sample size, the difference analysis was performed, setting the *P*-adjust <0.05 and |log2FC| >0.5. 1672 DEGs were obtained, among which 396 genes were down-regulated and 1276 genes were up-regulated. The results were visualized with a volcano map (**Figure 4A**) and a heat map (**Figure 4B**). Then signaling pathway enrichment for patients with PGK1 expression levels were analyzed using GSEA analysis. The analysis revealed that the high PGK1 expression was synergistic with endocytosis, leukocyte transendothelial migration, TNF signaling pathway, and PI3K-Akt signaling pathway, which were involved in various immune-related processes. Low PGK1 expression was related with antigen processing and presentation and Th1 and Th2 cell differentiation (**Figures 4C, D**).

Also, the genes correlated with PGK1 expression were explored using Pearson correlation, among which 710 genes were positively associated and 30 genes were negatively associated (**Figure 4E**). The correlation of 20 most relevant genes expression were shown (**Figure 4F**). Furthermore, the GO and KEGG enrichment analysis were employed to study the functional effects of PGK1. GO analysis revealed that PGK1-related genes were enriched in leukocyte degranulation, neutrophil activation involved in immune response, myeloid leukocyte activation, cytokine-mediated signaling pathway, and cellular response to interferon-gamma (**Figure 4G**). KEGG analysis disclosed that PGK1-related genes were mainly associated with leukocyte transendothelial migration, Fc gamma R-mediated phagocytosis, MAPK signaling pathway, chemokine signaling pathway, toll-like receptor signaling pathway, and T cell receptor signaling pathway (**Figure 4H**). Overall, the function of PGK1 was evidently associated with abnormal immune response.

### 3.4 Immune infiltration features were markedly distinct between high and low PGK1 sepsis group

**Figure 5:**
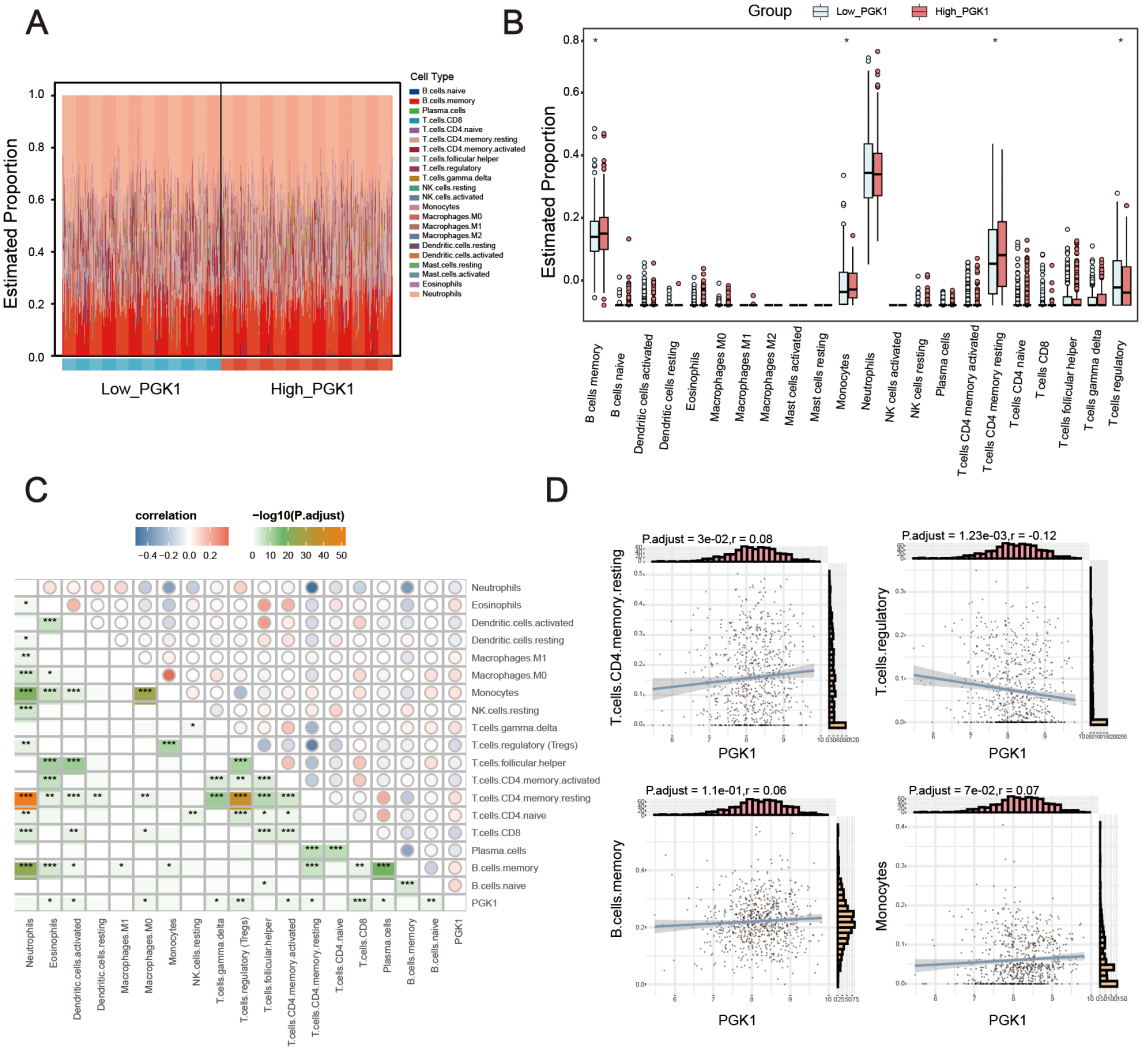
PGK1 was closely related with immune infiltration. **(A)** Stacked bar chart displayed the landscape of 22 types of immune cells in high_PGK1 and low_PGK1 group. **(B)** Violin diagram showed the difference in immune infiltration between the two groups. **(C)** Correlation matrix of PGK1 and immune cell proportions, the pie size indicates the degree of correlation. **(D)** Correlation between PGK1 expression and immune cells. *P < 0.05, **P < 0.01, ***P < 0.001.

Considering function enrichment analysis suggested that PGK1 was related to immune response, we further explored the relationship between PGK1 and immune cell infiltration. Using CIBERSORTx algorithm, we first analyzed the different abundance of immune cells between healthy controls and sepsis patients. The results showed the abnormal abundance of multiple immune cells in sepsis, among which a total of 3 kinds of cells increased and 3 kinds of cells decreased (**Supplementary Figure S1**, **Supplementary Table S1**). We also constructed the map of 22 immune cells in sepsis samples, and investigated the immune infiltration feature of high PGK1 group and low PGK1 group. Compared with the low PGK1 group, high PGK1 group presented higher memory B cells, monocytes, resting memory CD4 T cell and lower regulatory T cells (**Figures 5A, B**, **Supplementary Table S1**). Furthermore, it has been also revealed the relationship of PGK1 expression and the infiltration levels of 22 immune cells. The expression levels of PGK1 had significant correlations with a total of 10 kinds of immune cells, among which PGK1 expression was positively correlated with naive B cells, resting memory CD4 T cell, gamma delta T cells, M0 macrophages, eosinophils and negatively correlated with plasma cells, CD8 T cells, activated memory CD4 T cell, Tregs, activated dendritic cells (**Figures 5C, D**). These results further supported that the level of PGK1 might affect the immune activity of immune cells.

### 3.5 Characteristics of PGK1-related inflammatory molecules in sepsis

**Figure 6:**
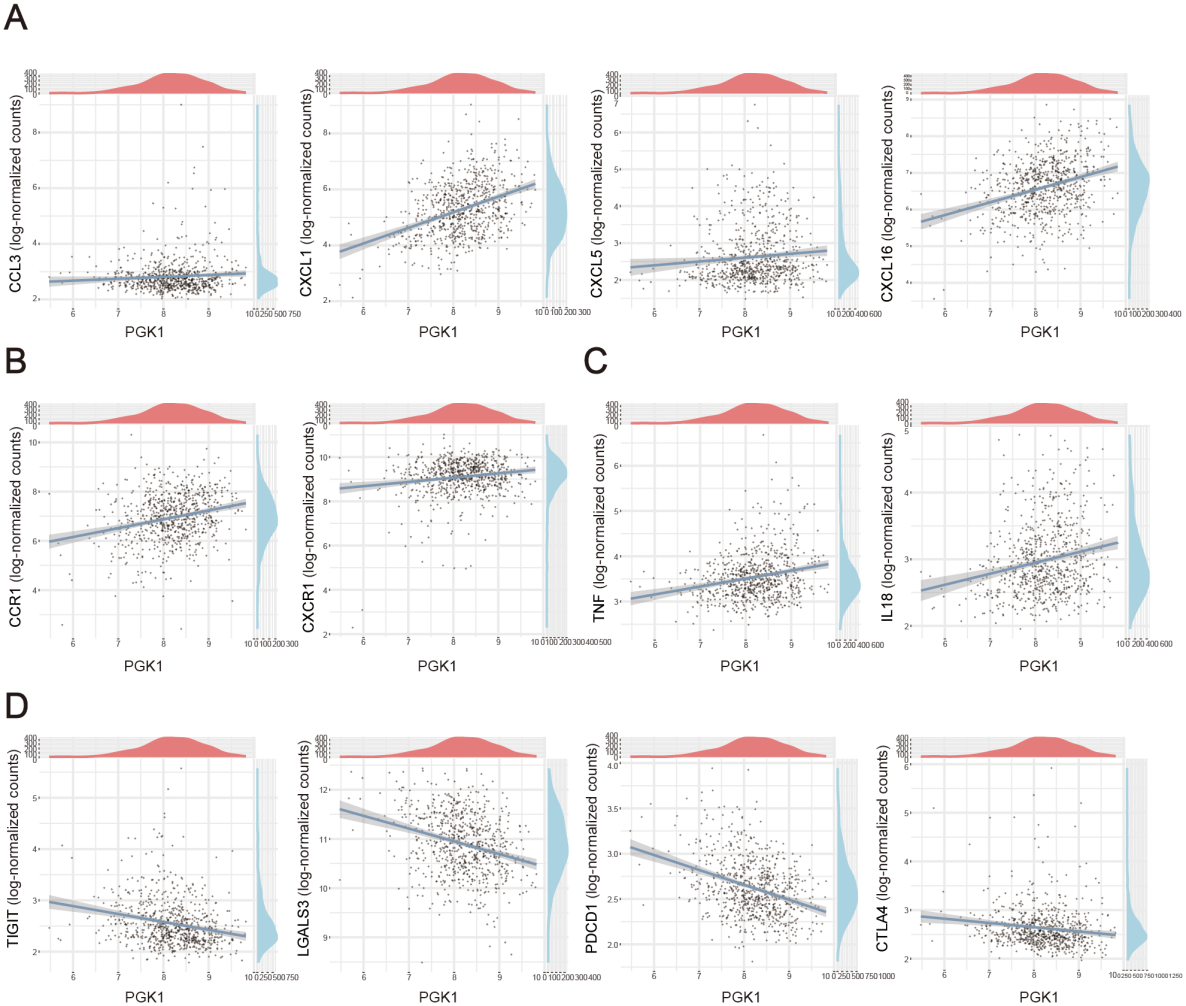
Correlation analysis between PGK1 expression and immunoregulation factors. Correlation between PGK1 and **(A)** chemokines, **(B)** chemokine receptors, **(C)** proinflammatory cytokines and **(D)** anti-inflammatory cytokines in sepsis patients.

Here, we also investigated the correlation of between PGK1 expression and various immune signatures including chemokines and cytokines in sepsis. Genes encoding chemokines and chemokine receptors were significant linked with PGK1 level. We observed that PGK1 level was positively correlated with chemokines, like CCL3, CXCL1, CXCL5, CXCL16 and chemokine receptors genes, including CCR1, CXCR1 (**Figures 6A, B**). Our data also observed the proinflammatory cytokines-encoded genes, including TNF, IL18 were positively correlated with PGK1 expression (**Figure 6C**). Expression of important anti-inflammatory factor-encoded genes such as TIGHT, LGALS3, PDCD1, CTLA4 were negatively correlated with PGK1 level (**Figure 6D**). As stated above, the sepsis patients exhibited a more inflammatory state compared to the healthy populations.

### 3.6 Aberrant PGK1 patterns in multiple immune cells of sepsis

**Figure 7:**
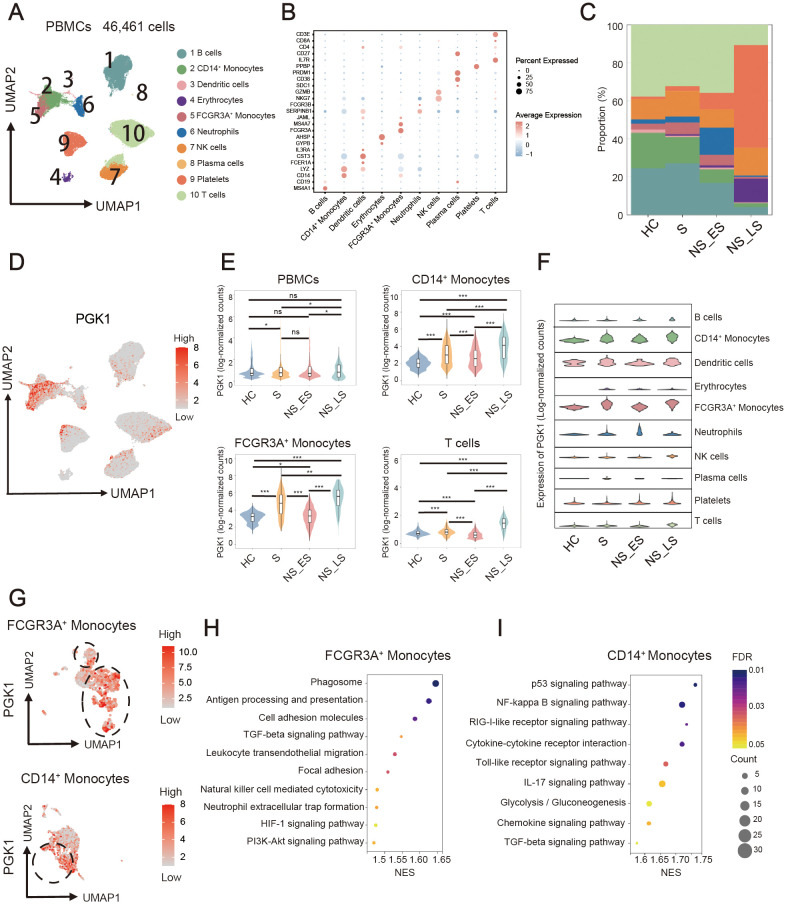
Single-cell transcriptomic profiling revealed the PGK1-related immune characteristic. **(A)** UMAP embedding plots of 10 cells by scRNA-seq profiles derived from sepsis patients and healthy people. **(B)** Expression distribution of cell identity specific RNA markers of sepsis cohort samples. **(C)** Proportions of cell clusters in healthy controls (HC), survivor (S), nonsurvivor, late-stage sepsis (NS LS), and nonsurvivor, early-stage sepsis (NS_ES). **(D)** UMAP plot represented PGK1 expression in distinct clusters. **(E)** Violin plots for the different proportion of cell subsets in HC, S, NS_LS, and NS_ES. **(F)** Abundance of major cell lineages in sepsis by scRNA-seq. **(G)** UMAP plot represented PGK1 expression in FCGR3A^+^ monocytes, and CD14^+^ monocytes. Bubble plots for GSEA enrichment of high_PGK1 and low_PGK1 **(H)** FCGR3A^+^ monocytes, and **(I)** CD14^+^ monocytes. NES, Normalized Enrichment Score. *P < 0.05, **P < 0.01, ***P < 0.001.

To reveal immune cell populations in sepsis patients, the graph-based clustering of UMAP was performed. According to the expression of canonical cell-type markers, we identified 10 major cell lineages including T cells, B cells, plasma cells, natural killer (NK) cells, CD14^+^ monocytes, FCGR3A^+^ monocytes, dendritic cells, neutrophils, erythrocytes, and platelets (**Figure 7A**). Expression distribution of cell identity specific RNA markers was showed (**Figure 7B**, **Supplementary Table S2**). Briefly, B cells were identified using the markers MS4A1 and CD19; CD14^+^ Monocytes were annotated with CD14 and LYZ; Dendritic cells were identified based on the expression of FCER1A, CST3, and IL3RA; Erythrocytes were marked by GYPB and AHSP; FCGR3A^+^ Monocytes were defined using FCGR3A and MS4A7; Neutrophils were annotated with JAML, SERPINB1, and FCGR3B; Natural Killer (NK) cells were identified using NKG7 and GZMB; Plasma cells were marked by SDC1, CD38, and PRDM1; Platelets were identified using PPBP; T cells were distinguished by IL7R, CD27, CD4, and CD8A. Then the patients were divided into survivor (S), nonsurvivor, late-stage sepsis (NS LS), and nonsurvivor, early-stage sepsis (NS_ES). Similar to the previous results (23), the proportions of T cells, B cells and monocytes in NS_ES group were significantly reduced, while platelets showed increased proportion (**Figure 7C**). We further investigated PGK1 expression in various cell clusters and different groups of patients, and found PGK1 had the highest expression in NS LS, followed by S, NS ES then HC in PBMCs, FCGR3A^+^ monocytes, CD14^+^ monocytes, and T cells (**Figures 7D, E**). Besides, PGK1 level in the NS_LS group was significantly increased compared with the S and NS_ES groups (**Figure 7E**), suggesting that PGK1 can potentially be a predictive marker for survival of sepsis. FCGR3A^+^ monocytes, and CD14^+^ monocytes from the sepsis patients presented the highest PGK1 expression compared to the other patients and other cell types (**Figure 7F**). Considering PGK1 was enriched in FCGR3A^+^ monocytes and CD14^+^ monocytes, we used UMAP visualization to identify clusters with high or low PGK1 expression of FCGR3A^+^ monocytes, and CD14^+^ monocytes (**Figure 7G**). GSEA enrichment analysis of differentially expressed genes between the two groups were performed. The analysis revealed that DEGs of FCGR3A^+^ monocytes mainly enriched in antigen processing and presentation, cell adhesion molecules, TGF-beta signaling pathway, leukocyte transendothelial migration, natural killer cell mediated cytotoxicity and neutrophil extracellular trap formation (**Figure 7H**). For CD14^+^ monocytes, these genes were enriched in pathways such as NF-kappa B signaling pathway, cytokine-cytokine receptor interaction, IL-17 signaling pathway, glycolysis/gluconeogenesis and chemokine signaling pathway (**Figure 7I**). Together, scRNA-seq analysis suggested the distinct PGK1 expression and immunomodulatory effects in various cell types of sepsis.

## 4 Discussion

This study elucidated the potential role of PGK1 in the pathogenesis of sepsis through the integration of microarray and single-cell RNA sequencing data. We demonstrated a significant upregulation of PGK1 in sepsis patients, underscoring its diagnostic value. The expression of PGK1 was found to be closely associated with various immune-related pathways and immune cells, suggesting its involvement in the immune response to sepsis and provide novel insights into the underlying regulatory mechanisms of PGK1.

PGK1, a key enzyme in the glycolysis process, plays a crucial role in cellular energy production by converting 1,3-bisphosphoglycerate into 3-phosphoglycerate and generating ATP (16). Beyond its metabolic role, PGK1 was also associated with the development of several immune-related diseases. In cancer research, PGK1 has been widely studied due to its frequent overexpression in cancer cells, which likely supported their rapid proliferation. For instance, high expression of PGK1 was significantly associated with poor prognosis in patients with non-small cell lung cancer (24). Similarly, in breast cancer patients, upregulation of PGK1 at both transcriptional and protein levels was associated with poor survival and prognosis (25). Moreover, PGK1 was known to impact the tumor microenvironment and immune evasion by regulating the expression of immune checkpoint molecules, as Li et al. (26) have reported. PGK1 role was also notable in inflammatory diseases. In rheumatoid arthritis, a study by Zhao et al. (27) found that PGK1 contributed to the activation of synovial cells and the release of pro-inflammatory cytokines. Similarly, in inflammatory bowel disease, higher PGK1 expression levels were linked to disease severity, suggesting its potential as an inflammatory biomarker (28). Our study’s finding of PGK1 upregulation in sepsis was consistent with these findings, indicating a broader role for PGK1 in immune system activation and disease progression.

The consistent upregulation of PGK1 across multiple GEO datasets and validation by qPCR and western blotting analysis in our study highlights its potential as a diagnostic biomarker for sepsis. The high AUC values obtained from ROC curve analysis demonstrate the excellent diagnostic performance of PGK1 in distinguishing sepsis patients from healthy controls. This finding may be significant considering the current limitations of sepsis diagnosis, which often relies on clinical criteria and non-specific markers. The ability PGK1 to distinguish between sepsis and non-sepsis conditions could aid in earlier identification and treatment of sepsis. Although there was no significant difference in overall survival between the high and low PGK1 expression groups in our study, patients with high PGK1 expression showed a trend toward poorer prognosis. We also analyzed the cell expression of PGK1 in different groups of sepsis patients by scRNAseq, and found that PGK1 level in the NS_LS group was significantly increased compared with the S and NS_ES groups, suggesting that PGK1 can potentially be a predictive marker for survival of patients with sepsis. This warrants further investigation to better evaluate the prognostic value of PGK1 in sepsis patients.

The enrichment of PGK1 in various immune functional pathways, as revealed by GSEA analysis, suggested its involvement in the immunopathology of sepsis. The association with leukocyte transendothelial migration, TNF signaling pathway and PI3K-Akt signaling pathway indicated that PGK1 may contribute to the dysregulated immune response observed in sepsis. Leukocyte transendothelial migration was an important immune and inflammatory response mechanism that enables white blood cells to reach the site of infection or tissue injury in time. TNF-α, produced by activated macrophages, initiates inflammation by binding to its receptors (TNFR1 and TNFR2), activating immune cells, and inducing the production of other inflammatory mediators (29). It also regulates cell survival and death, contributing to tissue damage (30). The balance of TNF-α is crucial for controlling the duration and intensity of inflammation, making it a key player in the inflammatory process (31). Previous study reported that inhibition of glycolysis leads to neutrophil immunosuppression during sepsis and may be controlled by downregulation of LDHA (lactate dehydrogenase A) mediated by the PI3K/Akt signaling pathway (19). These pathways are crucial for innate and adaptive immune responses, and their dysregulation can lead to excessive inflammation and organ damage.

The relationship between PGK1 expression and a spectrum of inflammatory mediators, including chemokines and cytokines, further corroborates its involvement in the inflammatory response during sepsis. CXCL1 and CXCL16 are chemokines that attract neutrophils and monocytes (32–34),s, while CCR1 is a chemokine receptor expressed on the surface of various immune cells (35). The recruitment of these cells is a critical step in the early stages of the inflammatory response, as they are responsible for phagocytosis and the release of pro-inflammatory cytokines (36, 37). The positive correlation with CXCL1, CXCL16, and CCR1 indicated that PGK1 may contribute to the recruitment of immune cells, such as macrophages and T cells, to the site of infection or inflammation. On the other hand, the negative correlation with LGALS3 and PDCD1 suggested that PGK1 may have a regulatory role in the balance between pro-inflammatory and anti-inflammatory responses. LGALS3 also known as Galectin-3, plays a crucial role in inflammation by directly binding to pathogens and influencing the function of cells in the innate immune system. Its ability to participate in various functions, including immune defense against pathogens, makes it an important player in the inflammatory response (38). PDCD1 (PD-1) is a protein involved in the regulation of apoptosis and the immune response. It has a diverse range of binding partners, which collaborate with other immune checkpoint proteins to promote immune suppression. The PD-1 and its ligands form an inhibitory pathway that mediates immune tolerance and maintains immune homeostasis (39). The downregulation of these genes in the presence of increased PGK1 expression may indicate a shift toward a more pro-inflammatory state, potentially leading to an imbalance in the immune response and exacerbating the inflammatory process.

The significant differences in immune cell infiltration patterns between high and low PGK1 expression groups further underscored the role of PGK1 in immune regulation. Sepsis patients with high PGK1 expression presented higher memory B cells, monocytes, resting memory CD4 T cell and lower regulatory T cells. Our data also implicated correlation between PGK1 expression and the infiltration of various immune cell types, including naive B cells, resting memory CD4 T cell, gamma delta T cells, M0 macrophages, eosinophils, plasma cells, CD8 T cells, activated memory CD4 T cell, Tregs, and activated dendritic cells, suggested a complex role for PGK1 in the immune response during sepsis. This correlation indicated that PGK1 may be involved in promoting pro-inflammatory immune responses, which were characteristic of the acute phase of sepsis. Liao et al. (16) investigated the role of PGK1 in immune metabolism. They found that PGK1 influenced the inflammatory response by regulating the production of IL-1β and IL-6. Lu et al. (40) discovered that in mice with myocarditis, the expression of glycolysis and PGK1 increased in heart CD4+ T and Th17 cells. Treatment with the PGK1 inhibitor NG52 resulted in reduced heart inflammation and fibrosis, as well as improved contractile function in mice. Furthermore, NG52 inhibited the activation and differentiation of CD4+ T cells, suggesting that PGK1 may play a key role in the metabolic regulation of T cell differentiation and autoimmune myocarditis. The infiltration of macrophages, neutrophils, eosinophils, and NK cells is a critical component of the innate immune response to infection, where these cells play roles in phagocytosis, killing of pathogens, and release of inflammatory mediators (41). The activation of CD4^+^ memory T cells and gamma-delta T cells, which are involved in the adaptive immune response, further amplifies the immune response by producing cytokines and facilitating the recruitment of other immune cells (42, 43). The negative correlation between PGK1 expression and the infiltration of activated NK cells and CD8^+^ T cells was intriguing, as both were cytotoxic effector cells of the immune system. Activated NK cells and CD8^+^ T cells play crucial roles in maintaining the normal function of the immune system by directly killing infected cells and regulating immune responses (44). Our study suggested that PGK1 may inhibit the activation of these cells, leading to a shift toward a more pro-inflammatory state, potentially exacerbating the inflammatory process in sepsis.

The scRNA-seq data provided valuable insights into the expression of PGK1 in different cell types in sepsis. The high expression of PGK1 in FCGR3A^+^ monocytes, and CD14^+^ monocytes, suggested its role in regulating immune cell function and metabolism. The role of PGK1 in promoting pro-inflammatory immune responses and inhibiting immune suppression in sepsis was consistent with its known function as a key enzyme in the glycolysis pathway (45). Glycolysis is the primary energy source for activated immune cells (46), and the upregulation of PGK1 in these cells would provide them with the energy needed for their activation and function. Furthermore, the correlation between PGK1 expression and the infiltration of various immune cell types in sepsis suggested that PGK1 may be involved in the regulation of immune cell recruitment and activation during the acute phase of sepsis. This regulation was crucial for the balance between pro-inflammatory and anti-inflammatory responses, which was essential for the resolution of the infection and the prevention of excessive tissue damage.

In addition, there is growing interest in targeting PGK1 due to its role in glycolysis, cancer metabolism, and other diseases. Some small molecules or inhibitors that target PGK1 have been identified in experimental studies, but they are mostly in early research phases (47, 48). Besides direct inhibition, alternative therapeutic strategies targeting PGK1 included RNA interference (RNAi) or CRISPR/Cas9 gene editing to downregulate PGK1 expression. We will also carry out further experiments in the future to explore the mechanism of PGK1 and the therapeutic effect of PGK1 inhibitors in sepsis, so as to develop more promising targeted therapies of sepsis. Our study still has several limitations. The sample size of our study was relatively small, and further validation in larger studies is needed to confirm the diagnostic and prognostic value of PGK1. Additionally, further investigation into PGK1 expression and potential function in various immune cells is warranted.

In conclusion, our study demonstrated that PGK1 serves as a novel diagnostic biomarker for sepsis, with potential implications for prognosis and immune regulation. Further research is needed to fully understand the role of PGK1 in the pathogenesis of sepsis and to explore its therapeutic potential.

## Data Availability

The datasets presented in this study can be found in online repositories. The names of the repository/repositories and accession number(s) can be found in the article/**Supplementary Material**.

## Supplementary material

Supplementary Figure 1 Landscape of 22 types of immune cells in sepsis and healthy people.

Supplementary Table 1 Proportions of 22 types of immune cells in GSE65682.

Supplementary Table 2 Cell-type markers for single-cell RNA sequencing.
