# Supplementary figures and images for: Effects of PGK1 on immunoinfiltration by integrated single-cell and bulk RNA-sequencing analysis in sepsis
Source: Front Immunol. 2024 Dec 6;15:1449975. doi: 10.3389/fimmu.2024.1449975 (PMC11659135; doi:10.3389/fimmu.2024.1449975)

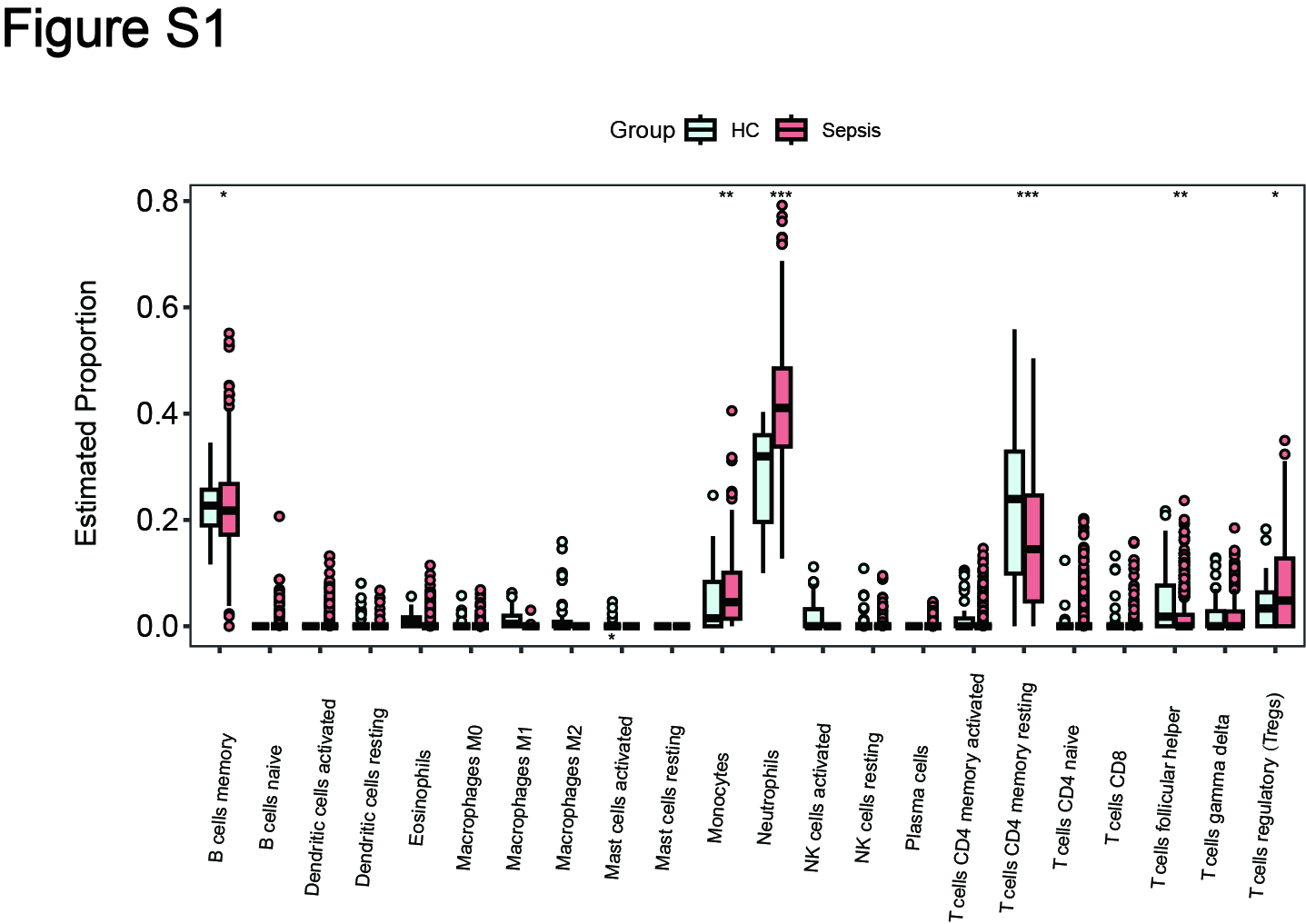

Supplement: Supplementary Figure 1 — Landscape of 22 types of immune cells in sepsis and healthy people. [file Image1.tif]
